# Enhancing clarity and methodological rigor in umbrella reviews

**DOI:** 10.1097/MS9.0000000000002536

**Published:** 2024-09-05

**Authors:** Abdullah, Humza Saeed, Muhammad Husnain Ahmad

**Affiliations:** a Rawalpindi Medical University, Rawalpindi, Punjab, Pakistan; b Tentishev Satkynbai Memorial Asian Medical Institute, Kant, Kyrgyzstan

*Dear Editor,*

Recent reports indicate that over ten systematic reviews and meta-analyses (SRMAs) are published daily, many are redundant and of low quality^1^. The proliferation of SRMAs necessitates addressing conflicting evidence and results among these reviews. Umbrella reviews fill this knowledge gap, positioned at the top of the evidence hierarchy, as they critically appraise multiple SRMAs on the same PICOS (Population, Intervention, Comparison, Outcome, Study Design) criteria. This approach directs readers to a comprehensive article rather than multiple SRMAs on the same topic.

SRMAs typically review high-quality studies, such as randomized controlled trials, followed by cohort and case-control studies. However, the quality of an umbrella review is contingent on the quality of the included SRMAs. Variations in methodologies, inclusion criteria, and quality among SRMAs can introduce heterogeneity and bias. Additionally, SRMAs are often cited in guidelines and used to shape clinical practice. If low-quality SRMAs are included in these guidelines, the standard of evidence-based medicine is compromised. Therefore, when both an SRMA and an umbrella review are available on the same PICOS criteria, the umbrella review should be preferred for evidence-based citation, as it consolidates the findings from multiple SRMAs and addresses overlapping studies. Thus, thorough and critical evaluation via umbrella reviews can significantly impact public health and clinical practice.

We read with great interest the article “Mental and Physical Health Morbidity among People in Prisons: an Umbrella Review” by Favril *et al.* ^2^, published in The Lancet Public Health. This high-quality methodological study highlighted the prominence of the umbrella review technique, which is the highest rank in the hierarchy of evidence. However, significant ambiguity remains regarding its proper conduct^3^. Reviewers and readers often struggle to interpret umbrella review results effectively. For example, Kons *et al.* ^4^ included 29 meta-analyses and provided a flow diagram with generic justifications for excluding 47 meta-analyses. However, they did not list the specific reasons for these exclusions, which contradicts AMSTAR-2 criteria^5^. We identified 27 pertinent meta-analyses^6–32^ that Kons and colleagues did not include, along with eight other relevant meta-analyses^33–40^ discovered simultaneously or after Kons *et al*.^4^’s submission. Additionally, it is unclear why one meta-analysis^41^ was chosen over others related to repeated-sprint training. This issue can be resolved by strictly defining inclusion and exclusion criteria for SRMAs.

Systematic reviews and meta-analyses vary widely, including comparisons, prevalence meta-analyses, single-arm meta-analyses, and diagnostic test accuracy studies. Comprehensive guidelines for conducting umbrella reviews that categorize them based on the types of meta-analyses included are lacking. Just as SRMAs follow Cochrane Handbook^42^ and PRISMA^43^ guidelines, RCTs follow CONSORT^44^ and observational studies follow STROBE^45^ guidelines, there should be specific guidelines for umbrella reviews. Currently, authors often use PRISMA guidelines designed for SRMAs for umbrella reviews, which could be improved.

Researchers frequently use the same risk of bias assessment (RoB) tools in umbrella reviews as in the incorporated SRMAs. This practice can introduce potential bias, as similar tools may produce consistent bias assessment patterns, diminishing the umbrella review’s overall quality. For example, if an SRMA uses the NOS scale^46^ for RoB of observational studies, we suggest that an umbrella review should use an alternate tool, such as ROBINS-I^47^, to provide a more robust critique. Quality issues and biases in primary studies can be compounded in an umbrella review, making them difficult to clarify. Surprisingly, Valkenburg *et al.* ^48^ did not even used any RoB tool neither for included SRMA nor for individual studies in each SRMA. This results in a lack of reliability in the overall results of the umbrella review.

Meta-analyses and umbrella reviews initially capture variability in exposure and outcome evaluations from the original research. While this variability cannot be eliminated, it can be managed through clearly defined inclusion and exclusion criteria. For example, Jepsen *et al.* ^49^ included “narrative reviews” with “systematic reviews” in their umbrella review. Thus, it is unwise to pool the outcomes of two different types of reviews under the same review where there is a huge difference between methodology and biases. This can create more complexity and less reliability in the results of their umbrella review. The reason lies in the lack of guidelines for umbrella review. The AMSTAR-2 tool, emphasizing 16 crucial categories, is frequently used for evaluating review quality^5^. It suggests avoiding subpar meta-analyses. However, for comprehensive analysis and debate, all reviews should ideally be included in an umbrella review, with methodological quality assessment occurring post-selection. To maintain a balanced approach, a macros tool (like the ROB-2 tool^50^ can be developed for the AMSTAR-2 tool to ease the RoB assessment for authors conducting such reviews. In this regard AI technology can be used to assist the development of more comprehensive RoB tools with accurate macros. Excluding studies preemptively can lead to information loss.

The Grading of Recommendations, Assessment, Development, and Evaluations (GRADE) technique is another method to assess the quality of evidence. Various methods have been proposed to evaluate the strength of evidence and determine the trustworthiness of each association. Some methods, based on credibility grading criteria proposed by Ioannidis and colleagues^51^, consider summary effect sizes, *P* values, sample size, number of events, heterogeneity, 95% prediction intervals, and tests of bias (e.g. small-study effects and excessive significance). These criteria can be integrated with other assessment techniques, like the GRADE methodology^52^, because they are categorized based on arbitrary cutoffs. However, they are limited by the potential absence of necessary information in the original research^52^. To enhance the quality of published articles, validated checklists and rigorous peer review are essential.

With the increasing number of network meta-analyses (NMA) being published, we can expect updated NMAs on the same topics, followed by umbrella reviews of these NMAs. The methodology for conducting umbrella reviews of NMA studies remains unexplored.

In conclusion, while umbrella reviews hold the potential for high-quality evidence synthesis, their methodological rigor and clarity need significant improvement. Adopting refined guidelines and robust quality assessment tools can help realize the full potential of umbrella reviews in informing public health and clinical practice. Such strategies can improve the evidence-based practice of medicine.

## Ethical approval

Ethical approval was not required for this editorial.

## Consent

Informed consent was not required for this editorial.

## Source of funding

The authors received no extramural funding for the study.

## Author contribution

All authors contributed to the conceptualization, literature review, drafting, editing, and reviewing of this editorial.

## Conflicts of interest disclosure

The authors declare no potential conflicts of interest concerning the research, authorship, and/or publication of this article.

## Research registration unique identifying number (UIN)

Not applicable.

## Guarantor

Not applicable.

## Data availability statement

Not applicable.

## Provenance and peer review

Not applicable.
